# ISOFORM-SPECIFIC FUNCTION OF C. ELEGANS TAU, PTL-1

**DOI:** 10.1093/geroni/igad104.3129

**Published:** 2023-12-21

**Authors:** Charlie Sconiers, Su-Hyuk Ko, Lizhen Chen

**Affiliations:** Howard University, Washington, District of Columbia, United States; University of Texas Health Science Center at San Antonio, San Antonio, Texas, United States; University of Texas Health Science Center at San Antonio, San Antonio, Texas, United States

## Abstract

As life expectancy increases with technological advancements, age-related dementia disorders, such as tauopathies, have become more rampant, impacting over 50 million people worldwide. Tauopathies are neurodegenerative diseases catalyzed by the dysregulation of tau protein in the brain. Alzheimer’s disease, the most common tauopathy, has impacted more than 6 million Americans in 2023. It has been speculated that within tauopathies, dysregulation only occurs in specific tau isoforms. After alternative splicing, two types of tau isoforms (one with three microtubule-binding repeats and one with four microtubule-binding repeats) may cause functional differences within the brain. A protein in C. elegans called PTL-1 was discovered to have similar microtubule-binding repeats in its isoforms. In this project, worms with two types of PTL-1 isoforms (PTL-1A and PTL-1B) and wild type worms were aged and consistently tested for their touch sensitivity and speed to document whether certain microtubule-binding repeats negatively impact function. It was found that PTL-1A worms lost speed and touch sensitivity at an expedited rate compared to the other two samples of worms. It was also found that some PTL-1A worms developed abnormal branches throughout their exons. These findings suggest that PTL-1A overexpression might cause morphological defects. These study results will assist in the development of tauopathy research and can be applied in evaluating how isoforms impact the genetic spread of neurodegenerative diseases.

